# Working in “NK Mode”: Natural Killer Group 2 Member D and Natural Cytotoxicity Receptors in Stress-Surveillance by γδ T Cells

**DOI:** 10.3389/fimmu.2018.00851

**Published:** 2018-04-24

**Authors:** Bruno Silva-Santos, Jessica Strid

**Affiliations:** ^1^Instituto de Medicina Molecular – João Lobo Antunes, Faculdade de Medicina, Universidade de Lisboa, Lisboa, Portugal; ^2^Division of Immunology and Inflammation, Department of Medicine, Imperial College London, London, United Kingdom

**Keywords:** γδ T cells, natural killer cell receptors, natural killer group 2 member D, natural cytotoxicity receptors, immunotherapy

## Abstract

Natural killer cell receptors (NKRs) are germline-encoded transmembrane proteins that regulate the activation and homeostasis of NK cells as well as other lymphocytes. For γδ T cells, NKRs play critical roles in discriminating stressed (transformed or infected) cells from their healthy counterparts, as proposed in the “lymphoid stress-surveillance” theory. Whereas the main physiologic role is seemingly fulfilled by natural killer group 2 member D, constitutively expressed by γδ T cells, enhancement of their therapeutic potential may rely on natural cytotoxicity receptors (NCRs), like NKp30 or NKp44, that can be induced selectively on human Vδ1^+^ T cells. Here, we review the contributions of NCRs, NKG2D, and their multiple ligands, to γδ T cell biology in mouse and human.

## Introduction

Natural killer cell receptors (NKRs) comprise various germline-encoded transmembrane proteins characterized for their capacity to regulate NK cell activation and homeostasis. This large family includes lectin-type receptors, natural cytotoxicity receptors (NCRs), and killer immunoglobulin receptors. The balance between activating and inhibitory signals derived from these receptors controls NK cell functionality. Besides their roles for NK cells, some NKRs, most notably natural-killer group 2 member D (NKG2D), have been known for long to be expressed by some subsets of T cells (1), including γδ T cells (2). In fact, nearly all human γδ T cells, and most mouse γδ T cells, express NKG2D. Importantly, we, and others, have shown that NKG2D is a key determinant of tumor cell recognition by murine intraepithelial γδ T cells (3, 4), as well as human peripheral blood (2, 5) and tumor-infiltrating (6) γδ T cells.

By contrast to NKG2D, NCRs were initially thought to be NK cell-specific (7), although this has changed particularly with the discovery of innate lymphoid cells (ILCs) (8). In fact, the acquisition of an NK-like phenotype and functionality was earlier reported on human intestinal intraepithelial lymphocytes (IELs), particularly in celiac disease (9, 10). The NCRs expressed on αβ IELs triggered interferon-γ (IFN-γ) secretion and degranulation (10), thus suggesting that IEL activation under inflammatory conditions favored the differentiation of “NK-like” effectors performing type 1 cytotoxic functions in an NKR-mediated (and TCR-independent) manner. We have recently built on this to show that human γδ T cells, specifically of the Vδ1^+^ subset, can be induced to express NCRs upon TCR plus IL-15 (or IL-2) stimulation *in vitro*, and these NCRs enhanced the capacity to target tumor cells of multiple origins, both *in vitro* and *in vivo* [(11, 12), and unpublished data].

In this mini-review, we will focus on the roles of NCRs, NKG2D, and their ligands for γδ T cell biology in mouse and human.

## NKG2D and its Ligands

The best-characterized activating NKR is NKG2D. NKG2D is a C-type lectin-like transmembrane receptor, which recognizes a range of different major histocompatibility complex class (MHC) I-related self-ligands induced or upregulated by a variety of cellular stress events, and notably on infected or transformed epithelial cells (ECs) (13). In mice, two isoforms of NKG2D exist, NKG2D-short (S) or NKG2D-long (L), while only the counterpart to the NKG2D-L isoform is expressed in human. The receptor functions as an activating receptor only through its association with signaling adaptor proteins, which are determined by the isoform of NKG2D expressed. NKG2D-S can associate with both DAP10 (recruits phosphatidylinositol 3-kinase) and DAP12 (activates tyrosine kinases Syk and ZAP70) while NKG2D-L is structurally incapable of associating with DAP12 and NKG2D-mediated signaling is mediated solely through DAP10 (14–16).

Engagement of NKG2D can trigger degranulation, cytotoxicity, and/or cytokine production—the distinct outcome of the receptor ligation may be explained by differential isoform and adaptor protein expression. Whereas, mouse CD8^+^ αβ T cells do not express DAP12 (and the exclusive NKG2D-DAP10 association serves as a costimulatory receptor), mouse epidermal γδ IELs constitutively express NKG2D-S, NKG2D-L, DAP10, and DAP12, and NKG2D ligation may trigger activity without TCR engagement (17).

**Figure 1:**
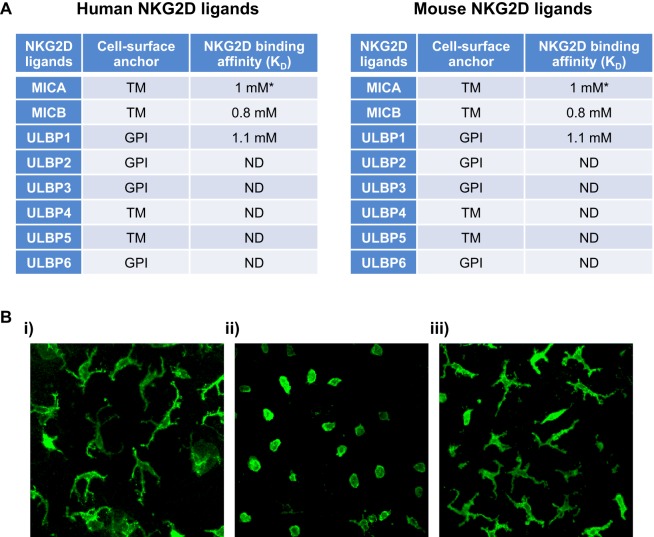
NKG2D ligands and a timely response to alteration in their expression by epidermal γδTCR^+^ intraepithelial lymphocytes (IELs). **(A)** Human and mouse NKG2D ligands, their cell surface anchor and their affinity to NKG2D are shown. **(B)** Representative confocal images of murine epidermal Vγ5Vδ1^+^ lELs in whole epidermal sheets following transgenic upregulation of Rae-1 under the involucrin promoter. (i) Single-transgenic and (ii) bi-transgenic mice were fed with doxycycline for 72 h, inducing expression of Rae-1 only in bi-transgenic mice (4). (iii) Mice with sustained expression of Rae-1 under the involucrin promoter (19). The images depict how acute expression of Rae-1 on epithelial cells induces morphological and activational changes in the neighboring IELs, whereas constitutive expression of Rae-l renders them hyporesponsive. Abbreviations: *allele-dependent NKG2D, natural killer group 2 member D; MIC, MHC class I-chain-related protein; ULBP, cytomegalovirus UL16-binding protein; Rae-1, retinoic acid early-inducible 1; MULT1, murine UL16-binding protein-like transcript 1; al, a2, and a3, analogous to the a1, a2, and a3 domains of MHC 1a proteins; TM, transmembrane protein; GPl, glycosylphosphatidylinositol-linked protein; ND, not determined.

Despite the different isoforms of NKG2D, the receptor is highly conserved with the receptors being 70% homologous between human and mouse, for example. NKG2D from one species can bind ligands from another (18). This is curious as the ligands are multiple and are both highly diverse in their amino-acid sequence, domain structure, membrane anchoring as well as exhibiting considerable allelic variation, and a wide range of receptor-binding affinities (Figure 1A). NKG2D ligands identified so far in humans include the MHC class I-chain-related proteins A and B (MICA and MICB) and six different UL16-binding proteins. In mice, three subgroups of NKG2D ligands have been identified: five isoforms of retinoic acid early-inducible 1 (Rae-1) proteins, one murine UL16-binding protein-like transcript 1 (MULT1), and three different isoforms of H60 proteins (Figure 1A). Why the NKG2D receptor is so promiscuous and engaging with so many ligands is not know, however, there are indications that not all ligands are functionally equivalent and that the diversity may allow for unique tissue-specific and contextual functions (1).

## NKG2D as a Critical Determinant of Mouse γδ T Cell Activation

Study of the NKG2D receptor is not only of huge academic interest, but clearly has therapeutic importance both within cancer, infection, and autoimmunity. Study of this receptor has also given us fundamental insight into γδ T cell biology. The capacity of murine tissue γδ T cells to act solely on alterations of autologous stress-antigens, such as those of the NKG2D receptor, and thus survey the “health-status” of a given EC has been termed lymphoid stress-surveillance (LSS) (4, 20, 21) (Figure 1Bi,ii). LSS highlights an important function of γδ T cells as afferent sensors of cellular dysregulations and as initiators of local and systemic immunity—a clear distinction from conventional αβ T cell biology. The activation of tissue γδ T cells during LSS *in situ* occurs seemingly without TCR stimulation (4). However, an alternative explanation could be that the TCR is constitutively engaged in the tissue as was suggested by an elegant study visualizing the γδ TCR continually signaling in the skin epidermis (22). The self-ligand recognized by the Vγ5Vδ1 TCR was not identified in this study, however, the implication of a possible constitutive TCR engagement in the tissue could be a predisposition to respond rapidly to stress-induced ligands recognized by co-stimulatory receptors, such as NKG2D. Whether substituting or synergizing with TCR signaling, these (co)stimulators are of pivotal importance in initiating and tuning the γδ T cell response. Interestingly, while epidermal γδ IEL activate and respond rapidly *in vivo* to transgenic acute upregulation of Rae-1 on ECs (4) (Figure 1Bi), prolonged constitutive expression of Rae-1 (19) renders them hyporesponsive and they remain in a resting state (Figure 1Biii). It is not clear how this tuning of the γδ IEL responsiveness is regulated according to the length of NKG2D-ligand expression, but the “beneficial autoimmunity” displayed during acute stress responses may be detrimental in chronicity and could be regulated by negative signals mediated by inhibitory NKRs, such as Ly49E and CD94-NKG2A (23). It would be of therapeutic value to understand when and how NKG2D-ligand hyporesponsiveness may occur as solid tumors, for example, can have long-term display of these ligands, which paradoxically could switch off resident tissue immune surveillance.

Natural killer group 2 member D clearly is a cytotoxic receptor and its engagement on γδ T cells has been shown in many murine studies to induce degranulation, cytotoxicity, and sometimes cytokine production (3, 17, 24). These studies all assessed γδ T cell function in isolation *in vitro* using γδ T cell lines. However, NKG2D ligands may not always function to generate cytotoxic responses *in situ* and the outcome of NKG2D receptor ligation is almost certainly context dependent. The different ligands have variable affinity for the receptor and may invoke differential responses. For example, the relatively low affinity ligand H60c is constitutively (and exclusively) expressed in the skin without evoking apparent cytotoxicity *in vivo* although it could induce cytotoxicity in γδ IEL cell lines *in vitro* (25). Moreover, while inducible expression of the high affinity Rae-1 ligand in the epidermis *in vivo* clearly activates the epidermal γδ IEL (Figure 1Bii) no overt cytotoxicity of the ECs was observed (4). Rather, a possible role for NKG2D-ligation in EC repair was indicated as this pathway was shown to induce potent expression of type 2 cytokines, particularly IL-13, from the γδ IEL, which functions to potentiate EC turnover, maintain an intact barrier, and thereby enhance resistance to carcinogenesis (26, 27). Further, Rae-1 transcripts were initially reported in mouse embryonic tissues and NKG2D ligands are also expressed in cells of the bone marrow (28, 29). Interestingly, MICA is also expressed by trophoblasts during normal human pregnancy, which may be sensed by uterine NK cells (which are not cytolytic). Together these observations suggest that NKG2D-ligand expression does not always evoke cytotoxicity *in vivo*, but may have an additional and relatively unexplored role in development and/or tissue repair.

## NKG2D-Dependent Activation of Human γδ T Cells

Most (60–95%) human peripheral blood γδ T cells express Vγ9Vδ2 TCRs that are uniquely activated by non-peptidic prenyl pyrophosphate antigens (phosphoantigens, PAg) such as isopenthenyl pyrophosphate, which is abundant in tumor cells; or (E)-4-hydroxy-3-methyl-but-enyl pyrophosphate, that is produced by bacteria and parasites [reviewed in Ref. (30)]. PAg-activated Vγ9Vδ2 T cells play important protective roles in infections, such as tuberculosis (31–34); and can kill a variety of tumor cell lines (35, 36).

Recent research has clarified how PAg may be “sensed” by Vγ9Vδ2 TCRs. This involves butyrophilin 3A1 (BTN3A1; also known as CD277), a B7 superfamily member that binds PAg in its intracellular B30.2 domain, which leads to significant conformational changes in the extracellular domains of the protein (37–43). Importantly, the effects of both agonist and blocking anti-BTN3A1 mAbs on Vγ9Vδ2 TCR transductants indicated that the TCR is necessary for the activation process (44, 45).

Besides TCR-dependent sensing of intracellular PAg accumulation, the discrimination between tumor and healthy cells by Vγ9Vδ2 T cells seemingly also involves NKG2D, which is expressed on the cell surface of nearly all Vγ9Vδ2 T cells, as it is on peripheral CD8^+^ αβ T cells. We have observed that NKG2D blockade reduces by circa 50%, the capacity of (PAg-activated) Vγ9Vδ2 T cells to target leukemic cells (as measured by apoptosis induction *in vitro*) (5). Moreover, when we looked for NKG2D ligands whose expression could account for leukemia cell recognition, we found ULBP1 to be the strongest candidate (5, 35). Consistent with this, the downregulation of ULBP1 impaired, whereas its overexpression enhanced, Vγ9Vδ2 T cell-mediated killing of leukemia/lymphoma cells (5). In independent studies, other NKG2D ligands have emerged as major determinants of tumor cell targeting by γδ T cells: ULBP4 in ovarian and colon carcinomas (46); and ULBP3 in B-cell chronic lymphocytic leukemia (CLL) (47). In the latter report, the critical γδ T cell subpopulation were Vδ1^+^ (rather than Vδ2^+^) T cells and Vδ1^+^ T cell counts as well as detectable/inducible ULBP3 expression both associated positively with disease control in CLL patients (47). Along the same lines, a recent study showed that ULBP1 and NKG2D expression associated (positively) with longer overall survival of gastric cancer patients (48). Thus, enhancement of NKG2D ligand expression, as achieved by bortezomib or temozolomide treatment of multiple myeloma (49) and glioblastoma multiforme (50) cells, respectively, may have important therapeutic potential, even if NKG2D ligand shedding may constitute an important immune evasion mechanism (51). On the other hand, tumor-directed recombinant ligands that engage NKG2D may also enhance tumor cell targeting, as recently documented against malignant B cells for bispecifics composed of CD20-binding and NKG2D ligand (MICA or ULBP2) domains (52).

The relative importance of NKG2D *versus* TCR stimulation of γδ T cells in human is still debated (53). Some studies reported the ability of Vγ9Vδ2 T cells to trigger effector responses through NKG2D stimulation alone (i.e., similarly to NK cells) (36, 54, 55). However, others have failed to show NKG2D-induced activation without simultaneous TCR stimulation (56). In this later case, NKG2D would function in γδ T cells like in CD8^+^ αβ T cells, i.e., as a costimulatory receptor accessory to the TCR. Future research should clarify whether the capacity to deploy NKG2D independently of the TCR varies between Vδ2^+^ versus Vδ1^+^ γδ T cells. The latter are preferentially found in mucosal tissues and can often be more abundant than Vδ2^+^ T cells within solid tumors (57). Unlike Vγ9Vδ2 T cells, Vδ1^+^ T cells do not recognize PAg; instead, intestinal epithelial Vδ1^+^ T cells were shown to bind MICA (and MICB) *via* a diverse set of Vδ1^+^ TCRs (58, 59), including on transfectants lacking NKG2D (60). Interestingly, our own data have identified a key difference between Vδ2^+^ and Vδ1^+^ T cells with regards to the ability to enhance their cytotoxic potential through the upregulation of a distinct class of NK cell receptors—the NCRs.

## Induced NCRs on Human Vδ1^+^ T Cells

**Figure 2:**
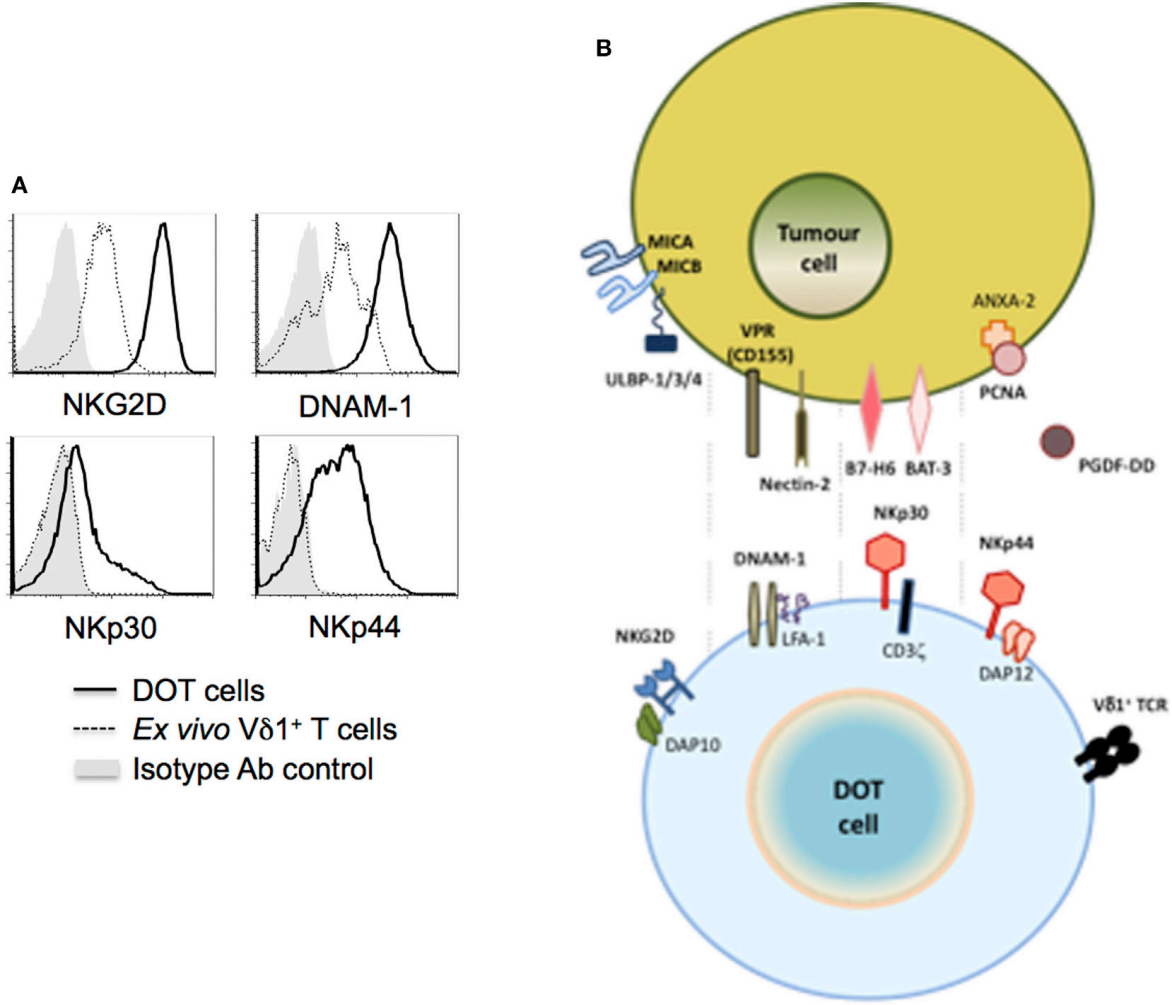
NK cell receptors and ligands for delta one T (DOT) cells. DOT cells are expanded/activated Vδ1^+^ T cells that upregulate NKG2D and DNAM-1 levels, and induce *de novo* NKp30 and NKp44 expression [**(A)** from Ref. (12)]. **(B)** The figure depicts putative ligands for those NK cell receptors known to be (over) expressed on tumor cells.

In contrast with NKG2D, NCRs were until recently thought to be NK-specific. However, reports on the acquisition of NCR expression by activated IELs (9, 10), and the subsequent identification of ILCs constitutively expressing NCRs, particularly NKp46 (8), have clearly demonstrated that NCR expression is not an exclusive property of NK cells. Although mouse γδ T cells seemingly do not express NKp46, we reported that the continued (>2 weeks) activation by TCR agonists (or mitogens-like PHA) in the presence of IL-15 or IL-2 induced NCR expression in a large fraction (>50%) of human γδ T cells (11). Interestingly, NCR induction was mostly restricted to Vδ1^+^ T cells, as Vδ2^+^ T cells failed to express any of the NCRs above background levels. And, in Vδ1^+^ T cells, the main induced NCRs were NKp30 and NKp44, with NKp46 limited to a smaller fraction (<20%) of activated cells. Antibody-mediated modulation and redirected lysis assays demonstrated the capacity of NKp30 and, to a lesser extent, NKp44, but not NKp46, to enhance Vδ1^+^ T cell cytotoxicity against tumor cell targets (11). Furthermore, NCR triggering increased IFN-γ expression in Vδ1^+^ T cells, consistent with their role in NK cells (61). Based on these findings, we have developed and established the pre-clinical proof-of-concept for a new (NCR^+^ Vδ1^+^) cellular product, delta one T (DOT) cells, for adoptive immunotherapy of cancer (12) (Figure 2). Moreover, we also demonstrated that this NKp30-mediated activation of Vδ1^+^ T cells was able to inhibit HIV viral replication, through the production of CCL3/MIP-1α, CCL4/MIP-1β, and CCL5/RANTES (62). These three CC-chemokines can inhibit viral replication by binding to CCR5, one of the primary co-receptors that HIV-1 uses for entry into CD4^+^ T cells. Collectively, these studies showed that the induced expression of NCRs on Vδ1^+^ T cells enhances their anti-tumor and anti-viral functions.

The acquisition of NCR expression by peripheral blood Vδ1^+^ T cells strictly requires strong TCR activation (11). This is consistent with previous work on human IELs, since the ability to upregulate NKp46 and NKp44 in the gut environment only occurred in effector T cells, and the expanded population of NCR^+^ αβ IELs in celiac disease displayed a highly restricted TCR repertoire indicative of oligoclonal expansion (10). Thus, the major current working hypothesis is that the expression of NCRs in T cells may derive from chronic activation *via* the TCR. This may allow pre-activated T lymphocytes to circumvent antigen-restricted responses, instead employing NCRs to respond continuously against “danger” or “stress,” which clearly fits the concept of LSS by γδ T cells (4, 20, 21).

In this context, it will be critical to identify the NCR ligands that are relevant to NCR^+^ Vδ1^+^ T cell biology, presumably associated with the processes of cellular transformation or viral infection. Thus, the hemagglutinin (HA) protein of the influenza and vaccinia virus binds NKp46 to stimulate NK cells to lyse virus-infected cells (63, 64). Conversely, a HCMV protein, pp65, was reported to inhibit NK cytotoxicity by dissociating the signaling chain, CD3ζ, from its complex with NKp30 (65).

Self-derived molecules have also been identified as ligands for NCRs, which underlies their capacity to target tumor and/or stressed cells. One of the first self-molecules identified to interact with NKp30 is the human leukocyte antigen-B-associated transcript 3 (BAT3) (66). The expression of this molecule on the tumor cell surface triggers NKp30-mediated killing and the production of IFN-γ and TNF-α. The anti-tumor role of BAT3 was confirmed *in vivo* by showing that peripheral blood NK cells were less efficient at clearing tumors when an anti-BAT3 blocking antibody was administered in mice.

A member of the B7 receptor family, B7-H6, was also shown to bind NKp30 (67). B7-H6 is present on cell surface of both primary tumor and tumor cell lines, while neither healthy nor stressed cells seemingly expressing it. Interestingly, while the presence of B7-H6 on the surface of tumor cells makes them susceptible to NKp30-mediated killing (67), the binding of B7-H6 to the alternative splice variant, NKp30c, reduces cytotoxicity and IFN-γ production, instead inducing NK cells to produce the immunoregulatory cytokine IL-10 (68). Moreover, the expression of the NKp30c isoform associated with poor clinical prognosis in gastrointestinal sarcoma.

As for NKp44, until recently its best-characterized ligand was an inhibitory molecule, proliferating cell nuclear antigen, that is commonly expressed by tumor cells, and strongly inhibited by NK cell cytotoxicity and IFN-γ production, likely *via* the inhibitory ITIM motif atypically present in the intracellular domain of NKp44 (69). However, a very recent study showed that NKp44 binds to platelet-derived growth factor-DD, a key promoter of tumor cell proliferation, epithelial–mesenchymal transition, and angiogenesis (70). The interaction provoked NK cell activation and the secretion of IFN-γ and TNF-α, which induced tumor cell growth arrest, including in *in vivo* cancer models. This may be a seminal finding in our understanding of how immune cells can recognize tumor cells. Future research should clarify the repertoire of NCR ligands that underlie the enhanced anti-tumor functions of NCR^+^ Vδ1^+^ T cells (Figure 2).

## Conclusion and Perspectives

Our current working model includes two stages of γδ T cell activation and target cell recognition: first, γδ T cells are activated by γδTCR ligands (many of which are still unknown); but then NKRs may play the key role in identifying stressed (transformed or infected) targets. This role is physiologically fulfilled by NKG2D (25), constitutively expressed by γδ T cells (3, 4); but therapeutically it can rely on induced NCRs, particularly for the human Vδ1^+^ T cell subset whose clinical potential is still to be realized (57). For these, the expression of both NKG2D and NCRs provides two functional layers of innate stress-surveillance, particularly of tumors. From a clinical perspective, we have established a clinical-grade protocol to differentiate NCR^+^ Vδ1^+^ T cells *in vitro*, from peripheral blood of cancer patients, toward the development of a new adoptive cell immunotherapy (“DOT cells”) (12). Of note, concomitant with TCR stimulation, IL-15 seems to be the key cytokine for NCR induction on Vδ1^+^ T cells (12), which is consistent with previous data on NK cells and IELs (61). Given that the gut is an IL-15-rich environment, it will be interesting to investigate the expression of NCRs on intestinal γδ T cells, especially since these compose a large fraction of the IEL compartment; and are highly enriched in Vδ1^+^ (compared to Vδ2^+^) T cells. Such future research should also clarify the enigmatic role of NKp46, since it was not evident from our studies on blood-derived Vδ1^+^ T cells (12); and address the potential relevance of NCRs for the reported regulatory functions of Vδ1^+^ TILs (71, 72).

Besides cancer, another potential application for NCR^+^ Vδ1^+^ T cells is the control of viral infection. Our demonstration that NKp30 engagement on *in vitro* activated Vδ1^+^ T cells is able to suppress HIV-1 replication through the production of CCL3/MIP-1α, CCL4/MIP-1β, and CCL5/RANTES, opens new avenues for the manipulation of γδ T cells in HIV-1 disease. This is particularly interesting because HIV-1 infection is characterized by a marked expansion of Vδ1^+^ T cells (73–75). This may be even more relevant in mucosal tissues, namely intestinal and cervical mucosa that are sites of HIV-1 entry, where Vδ1^+^ T cells are particularly abundant (76), while CD4^+^ T cells are strongly depleted (77). Of additional great potential is the use of NCR^+^ Vδ1^+^ T cells in CMV infection, given the well-established anti-CMV activity and long-term expansion of Vδ1^+^ T cells (78–81), including post-allogeneic stem cell transplantation (80–81).

In conclusion, we propose that NK cell receptor expression by γδ T cells contributes decisively to their role as a “bridge” between innate and adaptive immunity, both physiologically (*via* constitutive NKG2D) and therapeutically (through induced NCRs). We believe this entails great potential for future γδ T cell-based immunotherapies against viral infections or cancer.

## Author Contributions

BS-S and JS contributed equally to the inception and writing of the manuscript.

## Conflict of Interest Statement

BS-S is a co-founder and share holder of Lymphact—Lymphocyte Activation Technologies S.A. Jessica Strid declares that the research was conducted in the absence of any commercial or financial relationships that could be construed as a potential conflict of interest.
